# A PDM-based 128-Gb/s PAM4 fibre-FSO convergent system with OBPFs for polarisation de-multiplexing

**DOI:** 10.1038/s41598-020-58558-7

**Published:** 2020-02-05

**Authors:** Hsiao-Wen Wu, Chung-Yi Li, Hai-Han Lu, Qi-Ping Huang, Shi-Cheng Tu, Yong-Cheng Huang

**Affiliations:** 1 Department of Electronic Engineering, Tungnan University, New Taipei City, 222 Taiwan; 2 Department of Communication Engineering, National Taipei University, New Taipei City, 237 Taiwan; 3 Institute of Electro-Optical Engineering, National Taipei University of Technology, Taipei, 106 Taiwan

**Keywords:** Lasers, LEDs and light sources, Optical techniques, Optics and photonics, Engineering, Aerospace engineering, Electrical and electronic engineering

## Abstract

A polarisation-division-multiplexing (PDM)-based four-level pulse amplitude modulation (PAM4) fibre-free-space optical (FSO) convergent system with optical band-pass filters (OBPFs) for polarisation de-multiplexing is feasibly demonstrated for the first time. In a PDM scenario with PAM4 modulation, the transmission capacity of fibre-FSO convergent systems is enhanced four times with an aggregate channel capacity of 128 Gb/s (64 Gb/s PAM4/polarisation × 2 polarisations). With an OBPF, polarisation-tracking free de-multiplexing is attained by eliminating other optical carrier with orthogonal polarisation. An OBPF is a simple polarisation de-multiplexing scheme in which the polarisation-orthogonal carrier can be effectively de-multiplexed and the cross-polarisation interference can be nearly eliminated. Compared with traditional PDM-based fibre-FSO convergent systems with sophisticated polarisation-tracking mechanism and elaborate digital signal processing (DSP) approach, it reveals a noteworthy one with the advantage of simplicity. Through 25 km single-mode fibre transport and 500 m FSO link, sufficiently low bit error rate and qualified PAM4 eye diagrams are attained. This proposed polarisation-tracking free PDM-based fibre-FSO convergent system is notable because it not only incorporates the fibre backbone and optical wireless feeder, but it also simplifies the framework since complicated polarisation-tracking mechanism and DSP approach are not involved.

## Introduction

In the past few years, fibre-free-space optical (FSO) convergent systems have attracted considerable attention to connect fibre backbone and optical wireless feeder networks. Fibre-FSO convergence can make use of the traits of optical fibre and optical wireless communications, such as the naturally significant bandwidth of optical fibre and the unlicensed electromagnetic spectrum of optical wireless communication^1,2^. To incorporate the huge bandwidth of fibre backbone and the flexibility of optical wireless feeder, fibre-FSO convergent systems present a powerful scenario for providing present and emerging technologies, such as the broadband Internet, virtual/augmented reality, 4 K/8 K video streaming, and 5 G/Pre-6G mobile communications^3–7^. Compared with previous communication systems, fibre-FSO convergent systems can provide not only high transmission capacity but also satisfactory mobility.

Data center interconnect traffic is growing rapidly, along with the increasing popularity of Internet and mobile telecommunication applications. Such exponential growth of data-transmission loading has pushed the requirements for high transmission capacity. A polarisation-division-multiplexing (PDM) scenario has been explored to provide high transmission capacity^8–10^. In the PDM scenario, the transmission capacity of fibre-FSO convergent systems can be improved. Moreover, to further enhance the transmission capacity, four-level pulse amplitude modulation (PAM4) rather than none-return-to-zero (NRZ) modulation is adopted, provided that PAM4 modulation is an efficient approach to meet the targets of high transmission capacity^11–13^. In this study, a PDM-based 128-Gb/s PAM4 fibre-FSO convergent system over 25 km single-mode fibre (SMF) transport with 500 m free-space transmission is proposed and experimentally demonstrated. Two optical carriers (wavelengths) are polarized in different polarisations (*x*- and *y*-polarisations). The transmission capacity of fiber-FSO convergent systems is substantially multiplied by combining PDM scenario with PAM4 modulation. With optical band-pass filters (OBPFs) for polarisation de-multiplexing, the polarisation-orthogonal carriers (*x*-polarised and *y*-polarised carriers) are simply de-multiplexed for each polarisation. To our knowledge, this demonstration is the first one that constructs a high-speed fibre-FSO convergence with OBPFs to effectively de-multiplex the polarisation-orthogonal carriers. For traditional PDM-based lightwave transmission systems (two orthogonally polarised optical carriers with same wavelength), sophisticated polarisation-tracking mechanism and elaborate digital signal processing (DSP) approach are needed for polarisation de-multiplexing^14–21^. In 2018, Ding *et al*. achieved a polarisation-coherent scheme with adaptive polarisation control in wireless optical communication systems^14^. Li *et al*. demonstrated the delivery of a 54-Gb/s 8-quadrature amplitude modulation (QAM) W-band signal and 32-Gb/s 16-QAM K-band signal over 20 km SMF-28 and 2500 m wireless distance^15^. Hoang *et al*. achieved a 480-Gb/s single carrier signal with 60 Gbaud PDM-16-QAM over 80 km SMF with a bit-error-rate (BER) below the threshold of soft decision forward error correction^16^. Chagnon *et al*. proposed a DSP approach to process 4-D vectors and recover the intensity-modulation and phase-modulation information^17^. Wu *et al*. solved the polarisation-sensitive issue of radio-over-fibre systems by reprocessing polarisation-orthogonal lightwaves for upstream links^18^. Hou *et al*. attained a polarisation-stabilization control system based on polarisation-tracking mechanism and DSP approach^19^. Koch *et al*. established a 20-Gb/s PDM-RZ-DPSK transmission with 40 krad/s endless optical-polarisation tracking^20^. In 2010, Qian *et al*. demonstrated a 108-Gb/s multiple-input multiple-output orthogonal frequency-division multiple access passive optical network framework based on polarisation multiplexing and direct detection^21^. However, the polarisation-tracking mechanism and DSP approach will further complicate PDM-based lightwave transmission systems. For an actual deployment of a PDM-based lightwave transmission system, development of a polarisation de-multiplexing scheme with low sophistication is critically important.

We attainably constructed a PDM-based 128-Gb/s PAM4 fibre-FSO convergent system with OBPFs for polarisation de-multiplexing. In a PDM scenario with PAM4 modulation, the transmission capacity of fibre-FSO convergent systems is increased four times. With the adoption of polarization-independent OBPFs, the polarisation-orthogonal carriers are effectively de-multiplexed because they are modulated at different wavelengths. By removing other optical carrier with contrasting polarisation, polarisation-tracking free de-multiplexing is feasibly acquired. As for the traditional PDM-based fibre-FSO convergent systems, the polarisation-orthogonal carriers can’t be de-multiplexed by utilizing OBPFs because they are modulated at the same wavelength. Furthermore, two orthogonally polarised optical carriers with same wavelength will induce cross-polarisation interference and worsen the performance of fibre-FSO convergent systems. Through 25 km SMF transport and 500 m free-space transmission, competent BER performance and qualified PAM4 eye diagrams are achieved with an aggregate transmission capacity of 128 Gb/s (64 Gb/s/polarisation × 2 polarisations). The free-space link is increased to 500 m with the utilization of doublet lenses. Doublet lenses play vital roles in propagating laser beams through the free space^22,23^. A polarisation-tracking free PDM-based fibre-FSO convergent system is a promising convergence into which not only optical fibre backbone and optical wireless feeder networks are incorporated, but the traditional and complicated polarisation-tracking mechanism and DSP approach are not needed. This illustrated polarisation-tracking free PDM-based fibre-FSO convergence provides high transmission capacity and directs an access to accelerate the widespread applications through SMF transport with FSO transmission.

## Results

### Schematic diagram of multiple carrier generator with optical signal-to-noise ratio (OSNR) enhancement and the multiple carrier generator’s optical spectra before and after the OSNR enhancement setup

**Figure 1:**
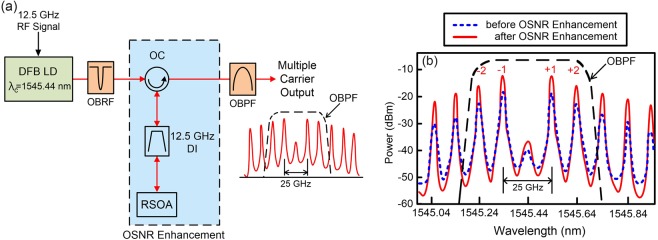
(**a**) The schematic diagram of the multiple carrier generator with OSNR enhancement. (**b**) The multiple carrier generator’s optical spectra before and after the OSNR enhancement setup.

Figure 1(a) presents the schematic diagram of the multiple carrier generator with OSNR enhancement. Multiple optical carriers are produced by directly modulating a distributed feedback (DFB) laser diode (LD) with a central wavelength of 1545.44 nm. The DFB LD is modulated with 12.5 GHz sinusoidal RF signal. The number of carriers is determined by the RF power level of the modulating signal on the DFB LD. For 12.5 GHz modulating signal with suitable RF power level, multiple coherent carriers will be produced with 12.5 GHz channel spacing. These optical carriers travel through an optical band-rejection filter, which is centered at 1545.44 nm and has a 0.03-nm 3-dB bandwidth, to filter out the zero-order carrier. Subsequently, they are enhanced by an OSNR enhancement setup and filtered by an OBPF, with a slope of 500 dB/nm, to pick up ±1 and ±2 optical carriers. The OSNR enhancement setup comprises an optical circulator (OC), a delay interferometer (DI) with 12.5 GHz free spectral range, and a reflective semiconductor optical amplifier (RSOA)^24,25^. The OC is employed to dispatch the produced optical carriers into the DI. With the DI, an RSOA is deployed to amplify and reflect the optical carriers. The DI serves as a comb filter to reduce each optical carrier’s valley power, and the RSOA serves as an optical amplifier to promote each optical carrier’s peak power. Accordingly, each optical carrier’s OSNR value is enhanced.

For a directly modulated lightwave transmission system, the electric field of modulating an RF signal can be depicted by way of following equation^26^:

1 $$E(t)=\sqrt{1+M\,\sin (2\pi {f}_{m}t)}\exp \{j\beta \,\cos [2\pi {f}_{m}t+{\varphi }_{f}({I}_{o},{f}_{m})]\}$$

where *M* is the modulation depth, *f*_*m*_ is the modulation frequency, *β* is the FM index, and *ϕ*_*f*_ is the phase delay which is determined by bias currents *I*_*o*_ and *f*_*m*_. A DFB LD with direct modulation will create intensity modulation (square root items) and phase modulation (exponent items) concurrently. The power difference among optical carriers can be reduced with an enhanced effect of phase modulation^27^.

The multiple carrier generator’s optical spectra before and after the OSNR enhancement setup are exhibited in Fig. 1(b). The figure shows that an approximately 6–9 dB OSNR value enhancement is acquired for each carrier as the produced coherent carriers pass through the OSNR enhancement setup. The connection between the *Q*-factor and the OSNR value is stated as^28^:

2 $$Q=\sqrt{\frac{1}{2}R\cdot OSNR\cdot \frac{\Delta {v}_{0}}{B}}$$

where *R* is the ratio of the peak photocurrent to the average, $\Delta {v}_{0}$ is the OSNR measurement bandwidth, and *B* is the receiver bandwidth. Clearly, from Eq. (2), the *Q*-factor is proportional to the square root of OSNR. Additionally, higher *Q*-factor value gives rise to better BER performance on the basis of the equation between the BER and *Q*-factor^29^:

3 $$BER=\frac{1}{2}erfc(\frac{Q}{\sqrt{2}})$$

A higher OSNR contributes to a higher *Q*-factor, leading to a better BER performance.

### Polarisation-orthogonal carriers’ optical spectra (*x*-polarisation and *y*-polarisation) with 64-Gb/s PAM4 signals and the optical spectra of the filtered (de-multiplexed) carriers

**Figure 2:**
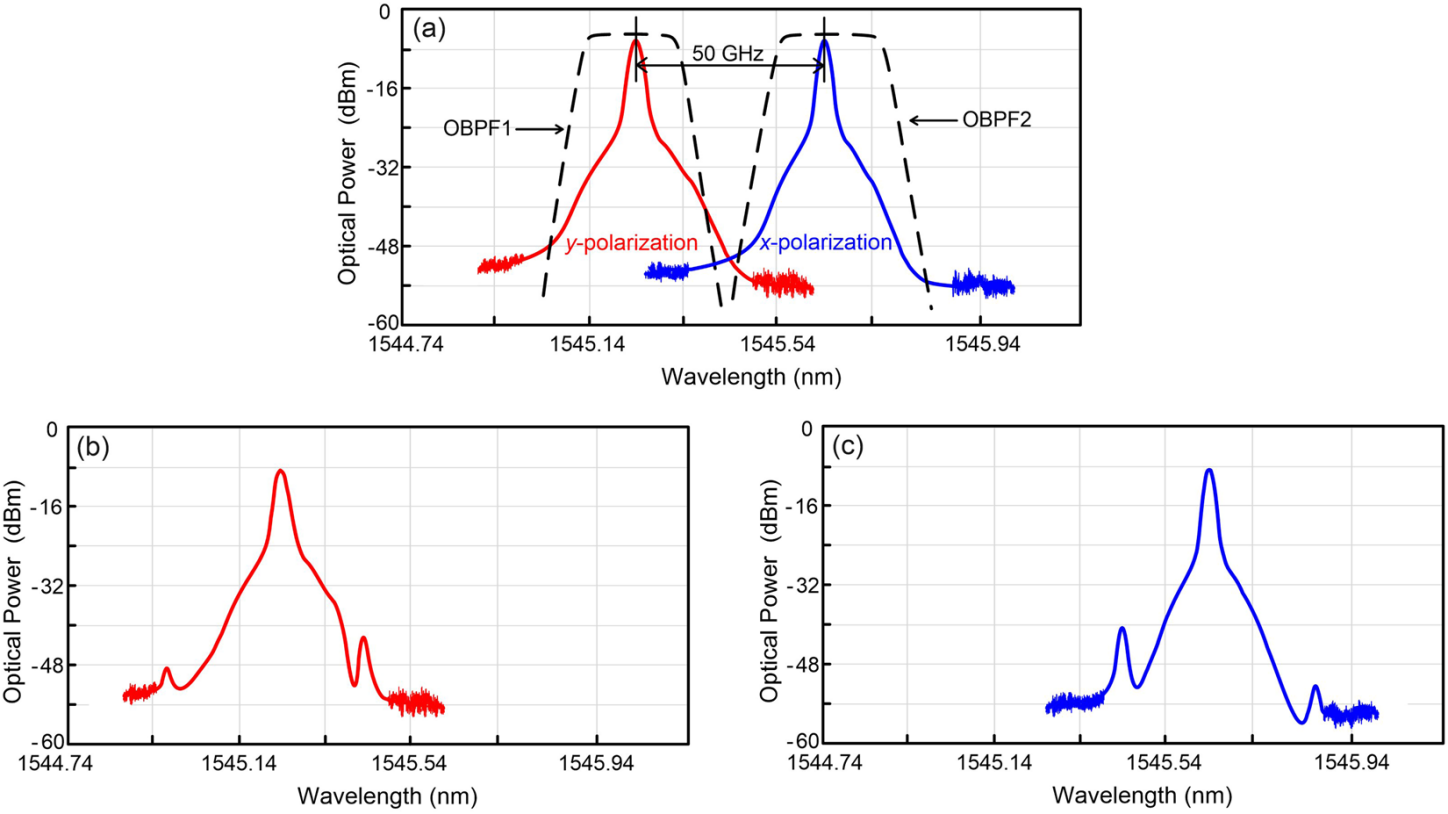
(**a**) The polarisation-orthogonal carriers’ optical spectra (*x*-polarisation and *y*-polarisation) with 64-Gb/s PAM4 signals. (**b**) The filtered carrier’s optical spectrum (*y*-polarisation). (**c**) The filtered carrier’s optical spectrum (*x*-polarisation).

Figure 2(a) displays the polarisation-orthogonal carriers’ optical spectra (*x*-polarisation and *y*-polarisation) with 64-Gb/s PAM4 signals. Moreover, the optical spectra of the filtered (de-multiplexed) carriers are presented in Fig. 2(b) (*y*-polarisation) and Fig. 2(c) (*x*-polarisation), respectively. With the use of OBPFs, the polarisation-orthogonal carriers are de-multiplexed from the PDM scenario. Polarisation-tracking free de-multiplexing is attained when other optical carrier with orthogonal polarisation is filtered out. The residual carrier with orthogonal polarisation is almost nonexistent after passing through an OBPF; therefore, the cross-polarisation interference will be trivially small. As for two optical carriers with parallel polarisations, distortions caused by self-polarisation interference will degrade the performance of the PDM-based fibre-FSO convergent systems due to the natural feature of two optical carriers with parallel polarisations. However, self-polarisation interference can be eliminated with the adoption of two optical carriers with orthogonal polarisations.

For three optical carriers with parallel/orthogonal polarisations, the corresponding square law detection can be calculated as^30^:

4 $$\begin{array}{ccc}{|{{S}}_{{x1}}+{{S}}_{{x2}}+{{S}}_{{y1}}|}^{2} & = & {|{{S}}_{{x1}}|}^{2}+{|{{S}}_{{x2}}|}^{2}+{|{{S}}_{{y1}}|}^{2}\\  &  & +2Re\{{{S}}_{{x1}}\bullet {{S}}_{{x2}}\}+2Re\{{{S}}_{{x1}}\bullet {{S}}_{{y1}}\}+2Re\{{{S}}_{{x2}}\bullet {{S}}_{{y1}}\}\end{array}$$

where *S*_*x*1_ and *S*_*x*2_ are optical carriers in *x*-polarisation, *S*_*y*1_ is optical carrier in *y*-polarisation, *S*_*x1*_·*S*_*x*2_ is the self-polarisation interference, and *S*_*x*1_·*S*_*y1*_ and *S*_*x*2_·*S*_*y*1_ are the cross-polarisation interferences. For two orthogonally polarized optical carriers (*S*_*x*2_ and *S*_*y1*_) with different wavelengths (proposed PDM scenario), given that no self-polarisation interference occurs and the cross-polarisation interference (*S*_*x2*_·*S*_*y1*_) is very small, good transmission performance can be acquired. For two parallel polarised optical carriers (*S*_*x1*_ and *S*_*x2*_) with wavelengths of *λ*_*1*_ and *λ*_*2*_, distortions caused by the self-polarisation interference (*S*_*x1*_·*S*_*x2*_) could be located at 2*λ*_*1*_ or 2*λ*_*2*_ (second-order harmonic distortion), *λ*_*1*_ ± *λ*_*2*_ (second-order intermodulation distortion), 3*λ*_*1*_ or 3*λ*_*2*_ (third-order harmonic distortion), 2*λ*_*1*_ ± *λ*_2_ or 2*λ*_*1*_ ± *λ*_2_ (third-order intermodulation distortion). All these distortions will worsen the performance of systems. As for two orthogonally polarised optical carriers (*S*_*x1*_ and *S*_*y1*_) with same wavelength (traditional PDM scenario), distortions caused by the cross-polarisation interference (*S*_*x1*_·*S*_*y1*_) will lead to performance degradation. In comparison with two parallel polarised optical carriers and traditional PDM scenarios, this proposed PDM scenario is worth employing because it can dramatically reject distortions produced by the self-polarisation and cross-polarisation interferences.

### BER performances of the PDM-based 128-Gb/s PAM4 fibre-FSO convergent system under different states

**Figure 3:**
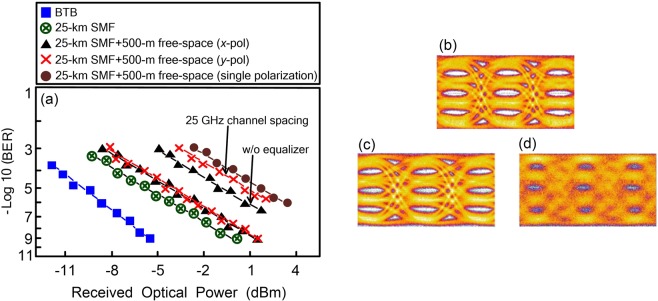
(**a**) The BER performances of the PDM-based 128-Gb/s PAM4 fibre-FSO convergent system in the states of BTB, through 25 km SMF transport, through 25 km SMF transport and 500 m free-space transmission (*x*- and *y*-polarisations), as well as through 25 km SMF transport SMF and 500 m free-space transmission (single polarisation). The eye diagrams of 64-Gb/s PAM4 signal in the scenarios of (**b**) BTB, (**c**) through 25 km SMF transport and 500 m free-space transmission (*x*-polarisation), and (**d**) through 25 km SMF transport and 500 m free-space transmission (single polarisation).

Figure 3(a) presents the BER performances of the PDM-based 128-Gb/s PAM4 fibre-FSO convergent system in the states of back-to-back (BTB), through 25 km SMF transport, through 25 km SMF transport and 500 m free-space transmission (*x*- and *y*-polarisations), as well as through 25 km SMF transport SMF and 500 m free-space transmission (single polarisation). Results show that the BER performances of *x*- and *y*-polarisations are almost exactly the same. Therefore, the BER performance’s impact in *x*- and *y*-polarisations is nearly identical. With 10^−9^ BER operation, a 6.6-dB power penalty emerges between the states of BTB and through 25 km SMF transport with 500 m free-space transmission (*x*- or *y*-polarisation). This 6.6-dB power penalty can be ascribed to the fibre chromatic dispersion associated with the 25 km SMF transport, atmospheric attenuation because of the 500 m free-space transmission, and coupling loss for coupling the laser light from the doublet lens 2 (doublet lens 4) to the ferrule of fibre^31,32^. Through 25 km SMF transport, a 5.5-dB fibre chromatic dispersion induced penalty exists. Over 25 km SMF transport, distortions caused by fibre chromatic dispersion worsens the BER performance on account of the natural feature of optical PAM4 signal with broadened linewidth. Over 500 m free-space transmission, an atmospheric attenuation of 0.5 dB occurs^33,34^. When laser light from doublet lens 2 (doublet lens 4) couples with the ferrule of fibre, a coupling loss of 0.6 dB occurs. Therefore, a link budget of 6.6 dB (5.5 + 0.5 + 0.6) emerges. The link budget of 6.6 dB exactly corresponds with the power penalty of 6.6 dB. The analysis results reveal that this demonstration suffices for the target of fibre backbone with optical wireless reach extender. Moreover, for two optical carriers with single polarisation (parallel polarisations), the BER value obviously degrades to 10^−6^. A poor BER performance of 10^−6^ is acquired as a result of self-polarisation interference and fibre chromatic dispersion. Distortions caused by self-polarisation interference degrades the BER performance. Through 25 km SMF transport, RF power reduction caused by fibre chromatic dispersion worsens the BER performance as well.

To verify the relation between the equalizer and the BER performance, we take away the equalizer and measure the BER values (*x*-polarisation) over 25 km SMF transport with 500 m free-space transmission. In the state without an equalizer, BER attains a value of 3.3 × 10^−7^. Whereas BER improves to 10^−9^ in the state with an equalizer (3.3 × 10^−7^ → 10^−9^). Equalizer is a type of amplifier, its amplification gain is a function of frequency. In here, the equalizer’s function is to enhance the amplitudes of high frequencies (18–30 GHz), compared to the amplitudes of low frequencies (DC–18 GHz), and bring on an increased SNR and an enhanced BER. Consequently, for this real-time PDM-based PAM4 fibre-FSO convergent system without adopting an equalizer at the receiving end, a BER value of ≤3.3 × 10^−7^ satisfies the communication criterion.

A PDM-based PAM4 fiber-FSO convergent system with a channel spacing of 25 GHz could be constructed. However, it is challenging to filter each optical carrier in such closely spaced optical carriers. The polarisation-orthogonal carriers can’t be effectively de-multiplexed by utilizing OBPFs due to a closed channel spacing of 0.2 nm (25 GHz). After passing through an OBPF, the residual carrier with orthogonal polarisation will bring on cross-polarisation interference, and thereby degrade the performance of PDM-based PAM4 fiber-FSO convergent systems. In the state with a channel spacing of 50 GHz, BER reaches a value of 10^−9^. Whereas BER degrades to 1.8 × 10^−6^ in the state with a channel spacing of 25 GHz (10^−9^ → 1.8 × 10^−6^; *y*-polarisation).

Figure 3(b–d) display the eye diagrams of the 64-Gb/s PAM4 signal in the scenarios of BTB, through 25 km SMF transport and 500 m free-space transmission (*x*-polarisation), as well as through 25 km SMF transport and 500 m free-space transmission (single polarisation). In the BTB scenario, with −5.2 dBm received optical power and 10^−9^ BER operation, open eye diagrams are acquired [Fig. 3(b)]. In the scenario of through 25 km SMF transport and 500 m free-space transmission, with 1.4 dBm received optical power and 10^−9^ BER operation, clear eye diagrams are acquired [Fig. 3(c)]. In the scenario of through 25 km SMF transport and 500 m free-space transmission (single polarisation), with 10^−6^ BER operation, turbid eye diagrams exist [Fig. 3(d)].

### BER performances of the PDM-based 128-Gb/s PAM4 fibre-FSO convergent system through 25 km SMF transport with various free-space links (*x*-polarisation)

**Figure 4:**
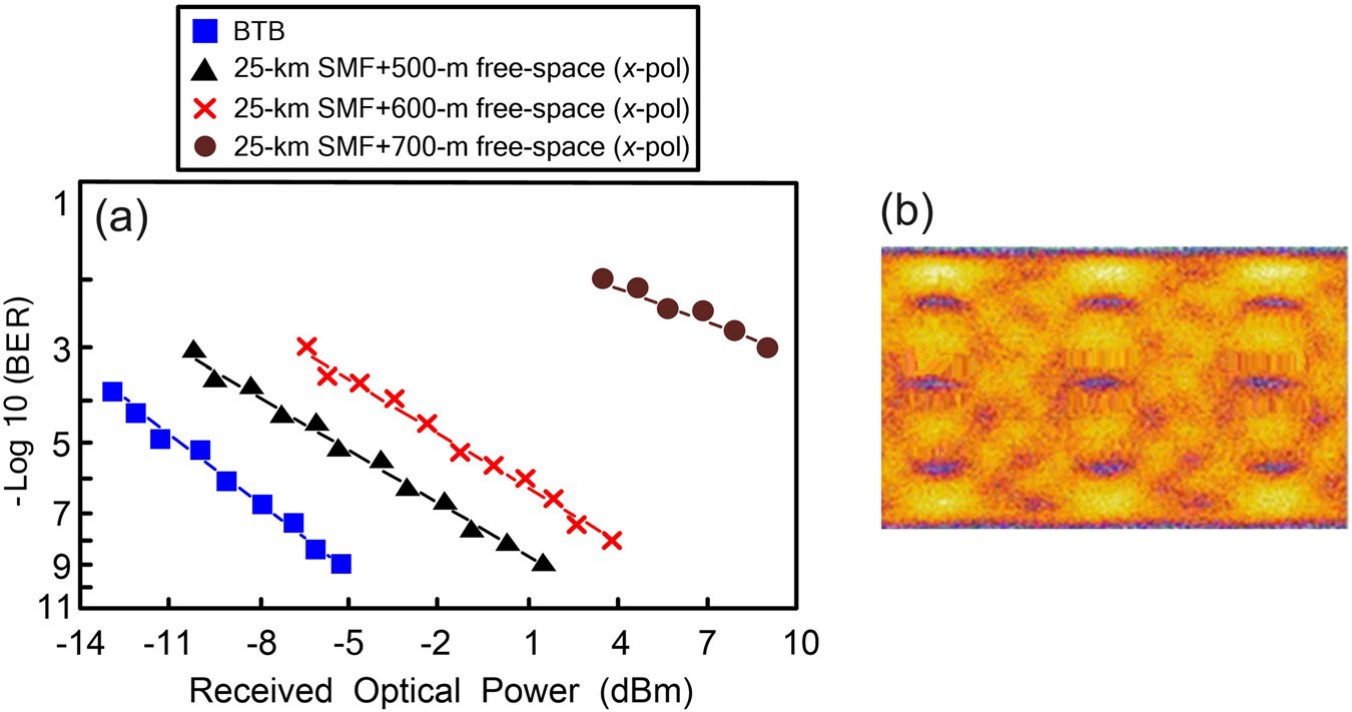
(**a**) The BER performances of the PDM-based 128-Gb/s PAM4 fibre-FSO convergent system through 25 km SMF transport with various free-space links (*x*-polarisation). (**b**) The eye diagrams of 64-Gb/s PAM4 signal in the scenario of through 25 km SMF transport and 700 m free-space transmission.

The BER performances of the PDM-based 128-Gb/s PAM4 fibre-FSO convergent system through 25 km SMF transport with various free-space links (*x*-polarisation) are presented in Fig. 4(a). As the free-space transmission distance reaches 500 m, a low BER value of 10^−9^ is acquired. As the free-space transmission distance extends to 600 m, the BER degrades to 10^−8^ due to further coupling loss compared to the 500 m free-space link. As the free-space transmission distance further extends to 700 m, the BER deteriorates to 10^−3^ because of greater coupling losses compared to the 500 m free-space link. The laser light sent from the transmitting side (doublet lens 1/doublet lens 3) will be expanded as it travels a 700-m free-space distance. With expanded laser light, the doublet lens (doublet lens 2/doublet lens 4) collects less light. The received optical power is noticeably reduced, resulting in poor BER performance. Additionally, in the scenario of through 25 km SMF transport and 700 m free-space transmission, close PAM4 eye diagrams are observed [Fig. 4(b)].

## Discussion

A set of doublet lenses is utilized to send laser light to the free space and to guide laser light to the fibre’s ferrule. The laser light’s transmission through the free space is extended to 500 m (50 m × 10) with the deployment of reflective mirrors on both sides. At the receiving end, a convergent scheme (doublet lens 2) should be implemented to couple laser light with fibre’s ferrule^35^. Doublet lens (AC508–150-C), with focal length/back focal length/diameter of 150 mm/150 mm/50.8 mm, is comprised of two lenses (concave and convex) that are paired together. The SMF’s numerical aperture is 0.14; therefore, the laser light’s diameter (*d*) can be calculated as:

5 $${d}=2\times (150\times 0.14)=42({\rm{mm}})$$

Because the laser light’s diameter (42 mm) is lesser than that of doublet lens 1 (50.8 mm), a free-space transmission with doublet lens 1 is feasible. Over an *L*-m free-space transmission, the laser light diameter (*d*_*L*_) must be lesser than that of doublet lens 2 (*d*_*L*_ < 50.8 mm) to comply with the field of view of receiver optics:

6 $${{d}}_{{L}}=\sqrt{{{d}}^{2}+{(2{\theta }L)}^{2}}=\sqrt{{42}^{2}+{(0.048{L})}^{2}} < 50.8$$

where *θ* is the beam divergence. *L* is computed as 595.4, indicating that the maximum free-space link is 595.4 m. The free-space link adopted in this work is 500 m (<595.4 m) to satisfy the maximum free-space link target. A set of fibre collimators with convex lenses might be adopted to substitute for a set of doublet lenses and establish a 500-m free-space link. Nonetheless, it is challenging to establish a 500-m free-space link utilizing a set of fibre collimators with convex lenses. The collimated laser light emitted from a fibre collimator with a convex lens will be spread as it travels over a certain free-space distance. In a free-space transmission of 500 m, the laser light’s diameter will be larger than that of doublet lens 2 (>50.8 mm). With large beam divergence, the convex lens with a fibre collimator at the receiving end will accumulate less transmitted light. An obvious decrease in the received optical power occurs, which is followed by poor BER performance.

Table 1 lists the optical spectral efficiency, power penalty (BTB state and that through 25 km SMF transport with 500 m free-space link), and BER under different scenarios. Although the optical spectral efficiency of our proposed PDM scenario is lower than that of the traditional PDM scenario, however, our proposed PDM scenario is worth adopting because it not only outperforms traditional PDM scenario due to a large reduction in cross-polarisation interference, but it also avoids the requirement of complicated polarisation-tracking mechanism and DSP approach. OBPFs adopted in the proposed PDM scenario remove the requirement for elaborate polarisation-tracking mechanism and sophisticated DSP approach. Given that the channel spacing between two optical carriers is 50 GHz, OBPFs with low filter slope and suitable cost are sufficient to meet the target. Additionally, our proposed PDM scenario outperforms the scenario of two parallel polarised optical carriers as well due to a reduction in self-polarisation interference.

**Table 1:** The optical spectral efficiency, power penalty, and BER under different scenarios.

Scenario	Proposed PDM	Traditional PDM	Two Parallel Polarised Optical Carriers
Metric			
Optical Spectral Efficiency	64 Gb/s/λ	128 Gb/s/λ	64 Gb/s/λ
Power Penalty	8.8 dB	9.4 dB	6.6 dB
BER	10^−9^	2.2 × 10^−5^	10^−6^

## Methods

### Schematic framework of the proposed PDM-based 128-Gb/s PAM4 fibre-FSO convergent system with OBPFs for polarisation de-multiplexing

**Figure 5:**
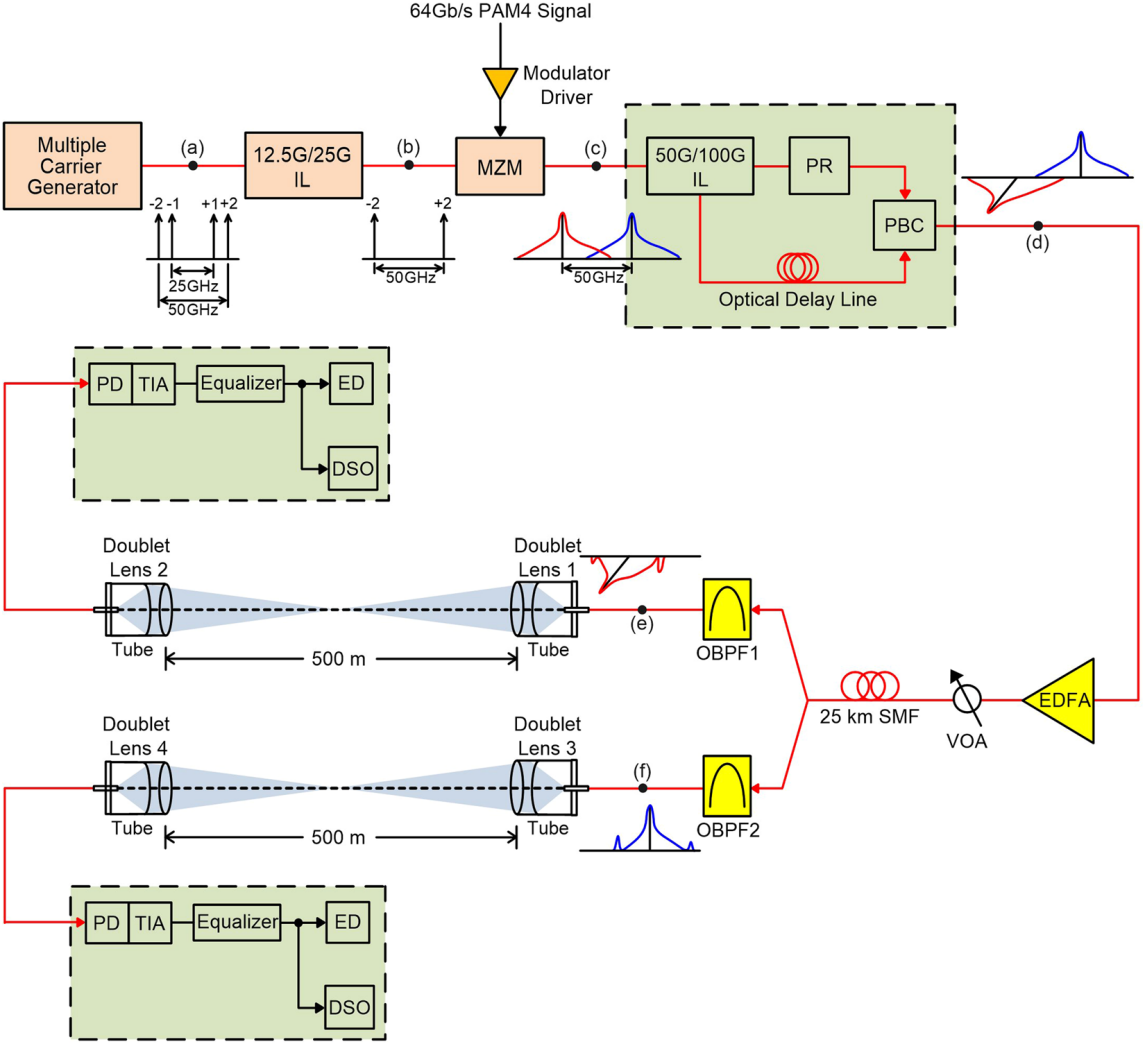
The schematic framework of the proposed PDM-based 128-Gb/s PAM4 fibre-FSO convergent system with OBPFs for polarisation de-multiplexing.

The schematic framework of the proposed PDM-based 128-Gb/s PAM4 fibre-FSO convergent system with OBPFs for polarisation de-multiplexing is presented in Fig. 5. A multiple carrier generator modulated with a 12.5-GHz RF signal is adopted to acquire multi-carrier with coherent feature and 12.5 GHz channel spacing. Optical signal with multiple optical carriers is modulated in the format of zero-order optical carrier suppression [insert (a)]. The multiple carrier generator’s output is then sent to a 12.5 G/25 G optical interleaver (IL) to separate odd and even optical carriers. Following the 12.5 G/25 G IL output with even optical carriers, two even optical carriers (−2 and +2) with 50 GHz apart [insert (b)] are supplied in a 40-GHz Mach-Zehnder modulator (MZM). Moreover, following the 12.5 G/25 G IL output with odd optical carriers, two odd optical carriers (−1 and +1) with 25 GHz apart are generated.

A 64-Gb/s PAM4 signal is adopted to modulate the MZM. Since that PAM4 linearity is important in the transmission performance, a high-linearity modulator driver is employed to linearize the electrical PAM4 signal. MZM’s output [insert (c)] is sent to a 50 G/100 G optical IL to separate two optical carriers. For the upper route, the optical carrier is inputted into a polarisation rotator to rotate the polarisation from *x*-polarisation to *y*-polarisation. Thus, two optical carriers are orthogonally polarized. For the lower route, an optical delay line is deployed to make up for the phase mismatch between the two routes. Subsequently, these *x*- and *y*-polarized carriers are combined utilizing a polarisation beam combiner [insert (d)]. After amplification by an erbium-doped filter amplifier and attenuation by a variable optical attenuator, the lights are transmitted over 25 km SMF transport. Then, the optical carriers are separated using an optical splitter. For the upper route, the *y*-polarized carrier with 64 Gb/s PAM4 signal is filtered by OBPF1 [inset (e)] and communicated over 500 m FSO transmission utilizing a set of doublet lenses. Over 500 m FSO link, the filtered *y*-polarized carrier is received and promoted by a photodiode (PD) with a trans-impedance amplifier (TIA) receiver, and equalized by an equalizer. After electrical equalization, BER measurement is performed in real-time using a high-sensitivity error detector (ED) and the PAM4 three-eye sampling approach^36^. Regarding typical DSP equalization algorithms, such as constant modulus algorithm (CMA) and decision-directed least mean square (DD-LMS) algorithm, adaptive combining techniques of soft and hard decision in a decision feedback equalizer structure with CMA and DD-LMS for blind acquisition and re-acquisition are required. Real-time BER measurement is attractive because it avoids the need of complicated offline DSP using Matlab. In addition, a digital storage oscilloscope (DSO) is deployed to take the eye diagrams of the transported 64-Gb/s PAM4 signal. For the lower route, the *x*-polarized carrier with 64 Gb/s PAM4 signal is filtered by OBPF2 [inset (f)] and communicated through 500 m FSO link utilizing a set of doublet lenses. Through 500 m FSO link, the filtered *x*-polarized carrier is detected and enhanced by a PD with a TIA receiver. After electrical equalization, BER measurement is executed in real-time with a high-sensitivity ED and the PAM4 three-eye sampling method. Also, a DSO is adopted to seize the 64-Gb/s PAM4 eye diagrams.
